# Civil Hospitals and the Care of Military Patients: Some United States' Problems

**Published:** 1918-05-11

**Authors:** 


### The Hospital

The Workers' Newspaper of Administrative Medicine and Institutional Life, Administration, National Insurance and Health.

No. 1666. Vol. LXIV. SATURDAY, MAY 11, 1918. PRICE ONE PENNY

### CIVIL HOSPITALS AND THE CARE OF MILITARY PATIENTS.

### Some United States' Problems.

Those of our readers who make a study of hospital progress, its claims and efficiencies, may remember that at the outbreak of the war in 1914 The Hospital dealt fully and at length during successive numbers with many matters included in the problem "Should the civil hospitals throughout the country be used for the care of military patients?" We ventured to advance a number of points embracing problems arising out of the war, the relation of military patients to civil hospitals, and the provision which must necessarily be made to secure adequate hospital accommodation and treatment for our warriors. The subject excited widespread interest. War has brought us many difficulties, the solution of which has engaged the combined wisdom and patriotism of the medical profession and many classes of people. At that time the United States were independent of the war, and free from war conditions. No one anticipated that America would be involved within its meshes, and, indeed, not only in the United States, but amongst neutral nations and in the countries of the combatants there were wide differences of opinion as to what part, if any, our cousins across the seas, who have now become our brothers in the war, would take.

A year ago the United States came to the momentous conclusion that they must strike home and strike hard to prevent the civilised world from being enslaved, downtrodden, and overrun by races inspired by greed, which have shown by their conduct that they are dead to every feeling which before the war decent people believed all responsible nations and their rulers would share and uphold. The United States found themselves at the outset surrounded by difficulties, with a host of momentous problems which called urgently for solution. There was, as with us in 1914, the call on the Medical Department from the Army to make provision for the hospital care, the medical and surgical treatment, the nursing, the commissariat, equipment—all that is imperative in the efficient modern clinical hospital of to-day—the ambulance and many other essentials. Where was this accommodation to come from, how were the medical and nursing staffs to be provided in adequate numbers and kept efficient, and what would best secure the wisest solution of the problems presented?

The first announcement of the U.S.A. Medical Department was that soldiers should not be sent to civil hospitals, "except in emergencies." Dr. S. S. Goldwater, chairman of the War Service Committee, American Hospital Association, in an interesting article in the April number of *The Modern Hospital*, demonstrates, however, that emergencies were expected to arise, for an official *questionnaire* was raised requesting the information concerning the capacity and equipment of civil hospitals, their willingness to accept such military patients as might be assigned to them, and the terms on which officers and enlisted men respectively would be received. It followed within a short period that invalid sailors and soldiers were admitted to civil hospitals side by side with other patients. By February 1918 it was ascertained that forty-six civil hospitals in New York State were caring for patients for either the Army or the Navy, a majority of them coming from the Navy; and in Philadelphia, Boston, and elsewhere the Navy has freely availed itself of the resources of civil hospitals.

Not unnaturally, a widespread discussion on the use of civil hospitals by the Government followed.

Dr. Goldwater insists that it presents five distinct problems: (1) the Army men's view that it is impossible for civil hospitals to care for their soldiers because civilian doctors have had no military training, are ignorant of medical needs, and are incapable of deciding, especially under conditions at or near the Front, whether a soldier physically incapacitated is fit to resume his place in the ranks. (2) That the number of sick in the cantonments is enormous, and even where these are situated in the neighbourhood of cities the civil hospitals are powerless to deal with the situation. In remoter localities this objection is declared paramount. (3) The advocates of the use of civil hospitals are thinking of another paramount point of view—*i.e.* the care of men incapacitated for service with the fighting forces, men who appear to be an encumbrance to the Army and a burden to its Medical Department. (4) Another opinion found expression in Canada by the action of a medical commandant there, who flatly declined to have anything to do with the first shipload of derelicts from France, on the ground that his services were more profitably employed in the preparation of men for the fighting line. He held that the care of physical wrecks returned from France was material for a civilian, not a military man. (5) Washington's official communication maintained, on the contrary, that the civil hospitals could not be counted on to relieve the Medical Department of the necessity of providing additional hospital facilities. The Army proposes, therefore, that men returned from abroad should be cared for in special military hospitals.

The question at issue which the National Administration has seriously to consider is whether the last proposal is the best way of caring for wounded Americans in the war. The War Service Committee of the American Hospital Association has proposed that the Medical Department undertake immediately a study of the conditions under which, and the extent to which, civil hospitals can best be utilised for Army purposes. This study, to be systematic, should include the consideration of the following problems: (*a*) The duplication of plant and equipment and the availability of military *personnel;* (*b*) medical administration, including especially the supply of a fit corps of physicians and their retention after the war; and (*c*) from the standpoint of nursing. It is pointed out that there are probably more than 8,000 soldiers being treated as in-patients in Canada. This is the present position, and a reference to the difficulties encountered in 1914 and their solution in this country, as fully set forth in The Hospital of August 15, 1914, and in subsequent issues, will afford interest and practical information.

Dr. Goldwater shows that at the time when the problems enumerated above first excited attention in the United States the British Hospitals Association had effected an agreement with the British Government for the treatment of disabled sailors and soldiers sent to the civil hospitals by the Ministry of Pensions. Both Associations are to be congratulated upon the important national service the war has rendered and will enable them to render to the Nations and the Empire. As many of our readers know, Dr. S. S. Goldwater is a man who has devoted himself for years to the mastery of hospital problems and hospital construction. He has a deservedly high reputation, and his services are greatly appreciated throughout the American hospital world. He concludes his article by urging Secretary Baker to provide that the study the Medical Department has been urged to make should be prosecuted vigorously and open-mindedly. Dr. Goldwater is a man of action, resource, sound judgment, and great experience, whose results have been fruitful in many directions. Our experience in this country and elsewhere, and a knowledge of the problems presented, with a keen appreciation of the difficulties to be faced, enforce the conclusion, that, the following position taken up by Dr. Goldwater is urgent and sound.

His article concludes: "The Army cannot afford to spend money for the construction of unnecessary buildings. It ought not to make a single serious blunder in the location of a 'special military hospital.' It cannot intend to erect costly sanatoriums to which soldiers returning from the other side will prefer not to go because of the distance of such sanatoriums from their homes. The Army cannot afford to establish 'special military hospitals' anywhere, for any purpose, unless it can place and keep in such hospitals competent staffs. It ought not to break up the clinical and nursing organisations of the civil hospitals for any reason less imperious than sheer military necessity. It cannot, and, unless we are greatly mistaken in Secretary Baker, in General Gorgas, and in the men with whom General Gorgas has surrounded himself, it will not, desire to adhere to any policy which runs counter to the principles of conservation or which tends to lower national efficiency during or after the war." *Experientia docet.*

